# Reductive aminations by imine reductases: from milligrams to tons

**DOI:** 10.1039/d2sc00124a

**Published:** 2022-04-07

**Authors:** Amelia K. Gilio, Thomas W. Thorpe, Nicholas Turner, Gideon Grogan

**Affiliations:** Department of Chemistry, University of York Heslington York YO10 5DD UK gideon.grogan@york.ac.uk; School of Chemistry, University of Manchester, Manchester Institute of Biotechnology 131 Princess Street Manchester M1 7DN UK nicholas.turner@manchester.ac.uk

## Abstract

The synthesis of secondary and tertiary amines through the reductive amination of carbonyl compounds is one of the most significant reactions in synthetic chemistry. Asymmetric reductive amination for the formation of chiral amines, which are required for the synthesis of pharmaceuticals and other bioactive molecules, is often achieved through transition metal catalysis, but biocatalytic methods of chiral amine production have also been a focus of interest owing to their selectivity and sustainability. The discovery of asymmetric reductive amination by imine reductase (IRED) and reductive aminase (RedAm) enzymes has served as the starting point for a new industrial approach to the production of chiral amines, leading from laboratory-scale milligram transformations to ton-scale reactions that are now described in the public domain. In this perspective we trace the development of the IRED-catalyzed reductive amination reaction from its discovery to its industrial application on kg to ton scale. In addition to surveying examples of the synthetic chemistry that has been achieved with the enzymes, the contribution of structure and protein engineering to the understanding of IRED-catalyzed reductive amination is described, and the consequent benefits for activity, selectivity and stability in the design of process suitable catalysts.

## Abstract 2

IRED-catalyzed reductive aminations have progressed from mg to ton scale, through advances in enzyme discovery, protein engineering and process biocatalysis.

## Graphical abstract

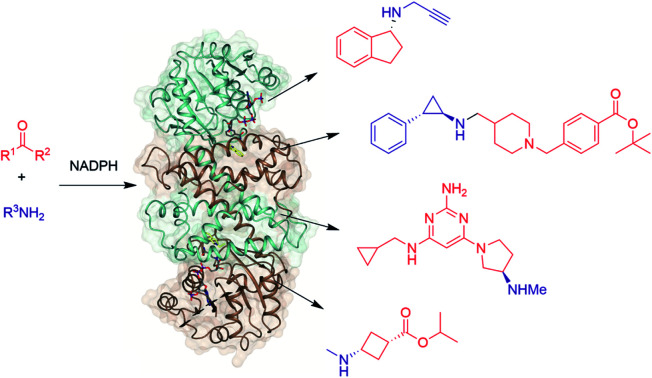

## Introduction

**Scheme 1:**
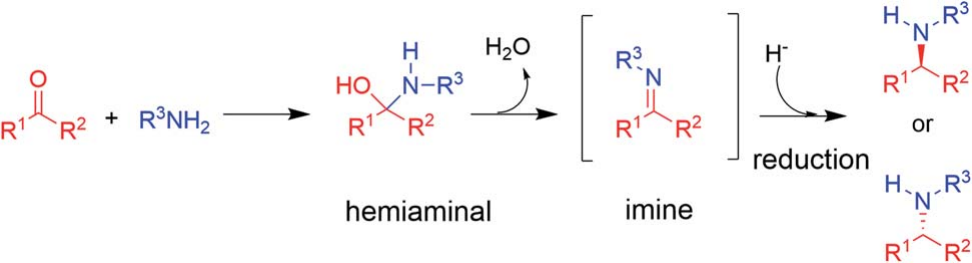
The asymmetric synthesis of chiral secondary amines *via* hemiaminal and imine formation by reductive amination (adapted from ref. 3).

The synthesis of chiral amines is of significant importance to the pharmaceutical and agrochemical industry as this group is widely distributed amongst the most used bioactive compounds in the world.^1,2^ Their synthesis is often accomplished through reductive amination,^3^ in which an amine is reacted with a ketone partner to form an intermediate hemiaminal, which then loses water to give an imine that is subsequently reduced to the corresponding amine (in the case of primary and secondary amine formations, the major focus of this perspective, Scheme 1).^3^

High optical purity is crucial for active pharmaceutical ingredient (API) manufacture and therefore a wide variety of reaction systems has been developed for asymmetric reductive amination to form chiral amines.

**Scheme 2:**
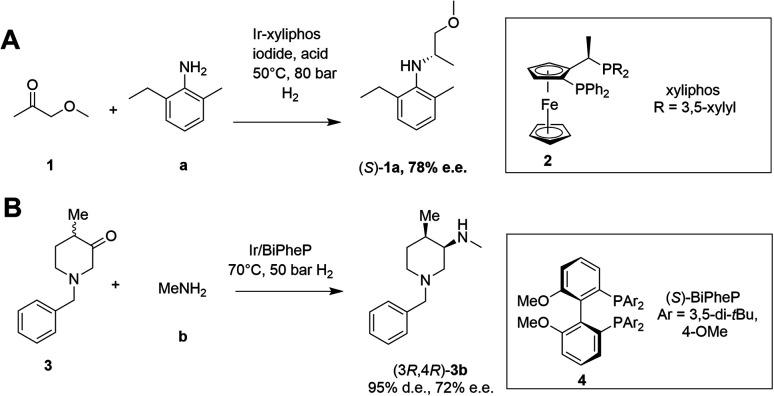
Examples of asymmetric reductive aminations catalyzed by chiral transition metal complexes. (A) Synthesis of (*S*)-metolachlor precursor;^11^ (B) synthesis of precursor of tofacitinib.^12^

Asymmetric reductive amination is often accomplished using organocatalysis^4–6^ or chiral transition metal (TM)-based catalysis.^7–10^ In an early example of the latter, Blaser showed that the precursor 1a of the herbicide (*S*)-metolachlor could be synthesized from methoxyacetone 1 with 2-methyl-5-ethylaniline a and hydrogen using an iridium catalyst generated *in situ* from 2, giving the amine product (*S*)-1a with 78% e.e. (Scheme 2).^11^

More recently, Verzijl and co-workers at Innosyn used a dynamic kinetic resolution-asymmetric reductive amination mediated by iridium catalyst 4 to synthesize a precursor of the rheumatoid arthritis treatment, tofacitinib.^12^ Using this reaction system, racemic ketone 3 was coupled with methylamine b using hydrogen, to give the (3*R*,4*R*)-amine product 3b in 86% isolated yield with 95% d.e. and 72% e.e. However, challenges remain for the general application of TM-catalyzed reductive amination, such as imperfect stereoselectivity, gaps in amine and ketone scope, as well as inhibition by amine derived intermediates.

**Scheme 3:**
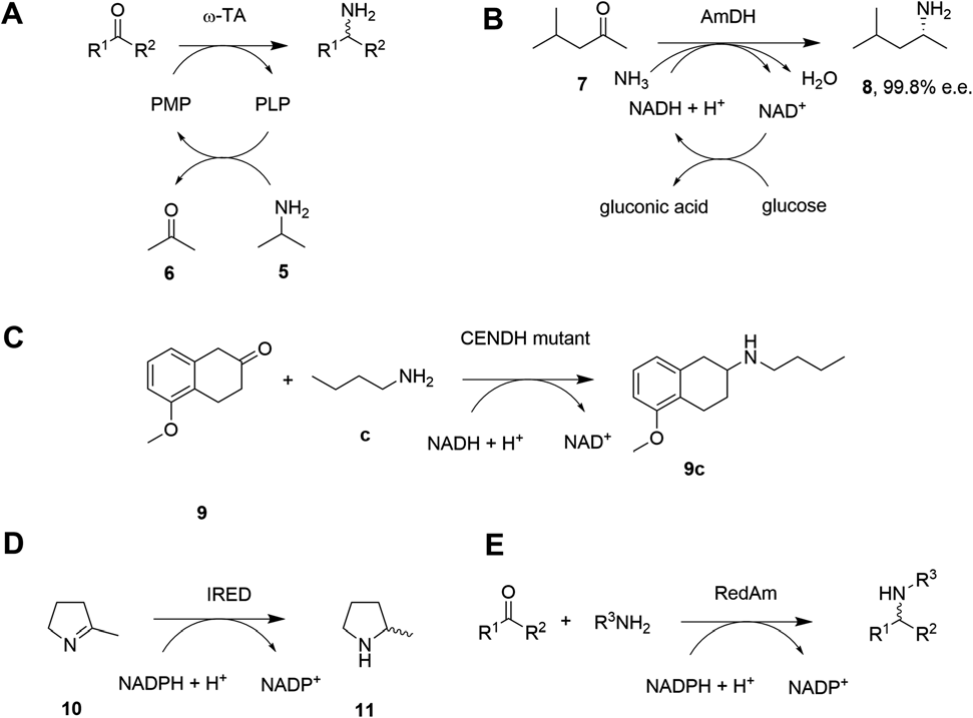
Biocatalytic amination of ketones by various enzymes. (A) ω-Transaminases (ω-TAs); (B) amine dehydrogenases (AmDHs); (C) opine dehydrogenases (OpDHs); (D) imine reductases (IREDs); (E) reductive aminases (RedAms).

Given the increasing pressure on the chemical industry to develop more sustainable routes to bioactive molecules, there has also been significant interest in developing biocatalytic methods for chiral amine production.^13,14^ Focusing on enzymes that perform reductive aminations, whether formal or direct, there are essentially five types that are of interest for their ability to transform prochiral ketones to chiral amines (A to E, Scheme 3 and Table 1). The first of these are ω-transaminases (ω-TAs, Scheme 3A),^15,16^ which convert ketones to amines through the use of an amine donor, such as l-alanine or isopropylamine 5. This amine donor is used by ω-TAs to aminate the cofactor pyridoxal phosphate (PLP) to pyridoxamine-5-monophosphate (PMP), with subsequent transfer of the amino group on PMP to the ketone substrate. ω-TAs have been the focus of a large amount of research in the last twenty years, including process improvements that combine protein and reaction engineering enabling large-scale industrial processes^17,18^ despite the need for excess amine donor to create a favourable reaction equilibrium. Additionally, a key limitation of ω-TA catalysis is that only primary amines can be synthesized and therefore secondary amine targets must be prepared through further elaboration.

**Table 1:** Types of enzyme that are used for the reductive amination of carbonyl compounds using ammonia or primary amine partners. Letters A to E correspond to reaction types illustrated in Scheme 3

	Enzyme	Mode of action	Cofactor	Will form primary amines	Will form secondary amines	References
A	ω-Transaminase (ω-TA)	Transfer NH_3_ from an amine donor *via* PLP/PMP cofactor	PLP/PMP	Yes	No	15 and 16
B	Amine dehydrogenase (AmDH; engineered or native)	Amination of ketones with NH_3_ followed by reduction using NAD(P)H	NAD(P)H	Yes	No, but with a few exceptions	19, 20, 29 and 30
C	Opine dehydrogenase (OpDH; engineered)	Amination of ketones with primary amines followed by reduction using NAD(P)H	NAD(P)H	No	Yes	35
D	Imine reductase (IRED)	Reduction of cyclic imines using NAD(P)H; also reduce imines formed in solution from carbonyls and amines	NAD(P)H	Yes, but not preferred	Yes, when amine is provided in excess	36 and 37
E	Reductive aminase (RedAm)	Reductive aminations of carbonyls with amines through catalysis of both imine formation and reduction	NAD(P)H	Yes, but not preferred	Yes, when amine is provided in 1 : 1 ratio with carbonyl	38

The second type of enzyme that catalyzes reductive amination is the amine dehydrogenases (AmDHs).^19,20^ AmDHs catalyze the direct reductive coupling of ketones with ammonia by forming an imine intermediate that is subsequently reduced by the biological hydride transfer agent NAD(P)H (Scheme 3B). Enzymes that catalyze the reductive amination of α-keto acids to α-amino acids (Amino Acid Dehydrogenases, AADHs) using this mechanism are well characterised, including, l-leucine^21^ and l-phenylalanine^22^ dehydrogenases (LeuDH and PheDH respectively). The simplicity of this route prompted protein engineering of LeuDH, targeted at altering the carboxylate binding pocket of the enzyme, and yielded a mutant for the enantioselective reductive amination of the aliphatic methyl isobutyl ketone (MIBK, 7) to give (*R*)-1,3-dimethylbutylamine 8 with 99.8% e.e.^23^ There are now numerous reports detailing the subsequent evolution and application of engineered AmDHs,^24–26^ which make use of other AADH scaffolds including PheDH and also an ε-deaminating l-lysine dehydrogenase (LE-AmDH),^27,28^ for the transformation of non-native substrates. More recently, a family of naturally occurring AmDHs was identified,^29,30^ that could transform simple ketones to amines using ammonia. These studies will prompt further screening and evolution efforts to expand the available substrate scope of AmDHs. The application of NAD(P)H-dependent AmDHs is attractive from an industrial perspective, given the simple nature of the amine donor, and also the use of nicotinamide cofactors, as technology for their implementation is well-established for the industrial exploitation of ketoreductase (KRED) enzymes.^31^ AmDHs are a valuable resource for the asymmetric amination of ketones, however, excluding a few recent exceptions,^32,33^ the product scope is limited to primary amines.

A third type of enzyme, opine dehydrogenases (OpDHs), which in Nature couple α-keto acids and an α-amino acids to form dipeptides,^34^ can also be engineered for the coupling of aliphatic ketones with primary aliphatic amines, in this case to form secondary amine products (Scheme 3C). For example, 9c could be synthesized from 5-methoxy-2-tetralone 9 and *n*-butylamine c using a variant of *N*-(1-d-carboxyethyl)-l-norvaline dehydrogenase (CENDH) obtained by directed evolution.^35^

Notwithstanding the discovery and application of engineered OpDHs, which does not appear to have been further explored by academic groups, the reductive amination of ketones to form chiral secondary amines, using alkylamine partners as co-substrates, remained both an attractive goal and a challenge for biocatalysis. In recent years this challenge has been met through the discovery and development of the fourth type of enzymes, the NAD(P)H-dependent imine reductases (IREDs),^36,37^ which catalyze the asymmetric reduction of cyclic imines such as 2-methylpyrroline 10 (Scheme 3D). IREDs can also enable the reductive amination of carbonyls when the amine partner is provided in large excess, recruiting the imine formed in solution for asymmetric reduction. In some cases the reductive amination between ketone and primary and secondary amine partners provided at a 1 : 1 molar ratio is achievable using the fifth type of enzyme, a sub-class of IREDs called the reductive aminases (RedAms, Scheme 3E). Since the description of this activity in 2017,^38^ applied on a 50–100 mg scale to laboratory proof-of-principle reactions, the technology has been very rapidly adopted by groups in academia and industry leading to multi-kilo,^39^ and even multi-ton^40^ chiral amine syntheses described in the public sphere. In this Perspective we trace the development of IRED technology for reductive aminations from the first small-scale transformations to industrial processes, highlighting the synergistic contribution of structure-guided enzyme evolution, with considerations of activity, selectivity, and also process suitability, to the development of large-scale reactions.

## Imine reductases (IREDs)

Imine reductases (IREDs)^36,37^ are NAD(P)H-dependent oxidoreductases that catalyze the asymmetric reduction of prochiral imines and iminiums forming chiral amines with high enantioselectivity (Scheme 3D). IREDs were first described by Nagasawa and co-workers from Gifu University in a patent filed in 2009,^41^ their activity being identified in strains of *Streptomyces* that catalyzed the asymmetric reduction of a model cyclic imine, 2-methyl pyrroline 10, to (*R*) or (*S*)-2-methylpyrrolidine 11.^42^ Isolation of these enzymes, followed by reverse genetics studies, led to the sequencing and heterologous expression of genes that encoded IREDs capable of either the (*R*)-^43^ or (*S*)-^44^ selective reduction of 10 when provided with NADPH. The identification of IRED sequences enabled the discovery of many thousands of genes of previously unknown function capable of imine reduction, and in the years up to 2017, several academic and industrial groups^45–54^ showed that panels of IREDs could be applied, either in cell-free form or expressed in whole cells, for the reduction of a broad range of substrates, such as monocyclic, bicyclic and even large tricyclic imines and iminiums, with excellent complementary stereoselectivity to (*R*)- or (*S*)-enantiomers.

**Fig. 1:**
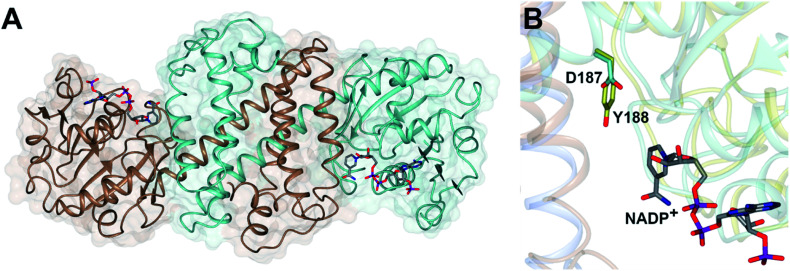
(A) Structure of dimer of (*R*)-selective IRED Q1EQE0 from *S. kanamyceticus* (PDB 3ZHB)^45^ with monomers (A) and (B) shown in brown and blue respectively; (B) detail of active site clefts in 3ZHB and (*S*)-selective IRED from *Bacillus cereus* (4D3F)^53^ showing cofactor NADP^+^ and residues D187 and Y188 from the respective enzymes.

The first published structures of IREDs^45,49,53,54^ permitted insights into their broad substrate scope, and also informed mutational studies that looked to provide the first information on possible mechanisms of catalysis. The IRED fold, typified by the enzyme Q1EQE0 from *Streptomyces kanamyceticus*,^45^ is shown in Fig. 1A. IREDs are dimers, constructed of two monomers each with an N-terminal Rossmann domain connected to a helical bundle C-terminal domain by a long interdomain helix. A domain sharing arrangement dictates that the active site is formed between the N-terminal domain of one monomer and the C-terminal domain of its partner, resulting in a large cleft that accommodates the nicotinamide cofactor and the substrate. Structures of IREDs that feature more than one dimer in the crystallographic asymmetric unit show that the cleft can exist in either more open or more closed states, the latter providing a hydrophobic environment for substrate binding.^45^

**Fig. 2:**
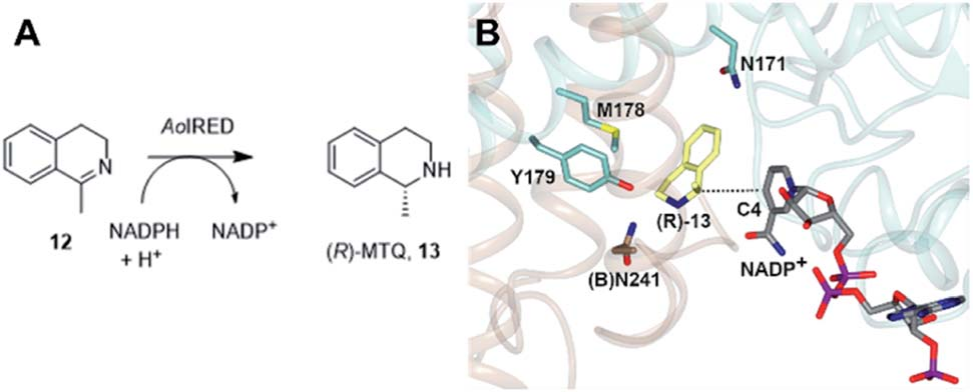
(A) Reduction of 1-methyl-3,4-dihydroisoquinoline 12 to (*R*)-MTQ 13 by *Ao*IRED^54^ (B) detail of active site of *Ao*IRED in complex with NADP^+^ (5A9S) and (*R*)-MTQ (5FWN). Residues are from monomer (A) unless marked as (B). The trajectory for delivery/acceptance of hydride from/to the C4 atom of NADP^+^ is shown with a dashed black line.

One characteristic of the active sites is a hydrophilic amino acid residue, in most cases, an aspartate or tyrosine, which projects from the ceiling of the active site pocket towards the nicotinamide ring of the cofactor (Fig. 1B). Enzymes featuring Asp^43^ or Tyr^44^ were shown to be (*R*)- or (*S*)-selective for the reduction of 2-methylpyrroline respectively. While the role of these residues in IRED catalysis has yet to be determined unambiguously, their mutation in (*R*)-^45^ or (*S*)-^48^ selective IREDs to alanine led to a marked decrease in activity. A structure of the enzyme from *Amycalotopsis orientalis* (*Ao*IRED), in complex with the amine product (*R*)-1-methyltetrahydroisoquinoline ((*R*)-MTQ) 13 (Fig. 2A) of the reduction of prochiral 1-methyl-3,4-dihydroisoquinoline 12,^54^ showed that the product was oriented in the active site in such a way that the electrophilic carbon of the substrate imine should be well positioned to receive hydride from the C4 atom of the nicotinamide ring of the cofactor from one face, giving the experimentally observed (*R*)-configuration in the product (Fig. 2B).

These and other structures suggested that the binding site was particularly suited to the reduction of hydrophobic cyclic imines and showed how a prochiral substrate could be positioned such that hydride delivery occurred predominantly at one face of the C=N bond.

## IREDs enable the synthesis of acyclic secondary amines

Despite the significant advances in chiral amine synthesis using IRED-catalyzed imine reduction, the technology was limited to cyclic imines that are sufficiently stable in aqueous solution for direct reduction to cyclic amines. However, the discovery that IREDs possessed broad substrate scope, high activity and stereoselectivity, as well as negligible activity toward the reduction of ketones, prompted researchers to ask whether, in addition to cyclic imines, the activity of IREDs could be expanded to include the formation and reduction of acyclic C=N bonds.

**Scheme 4:**
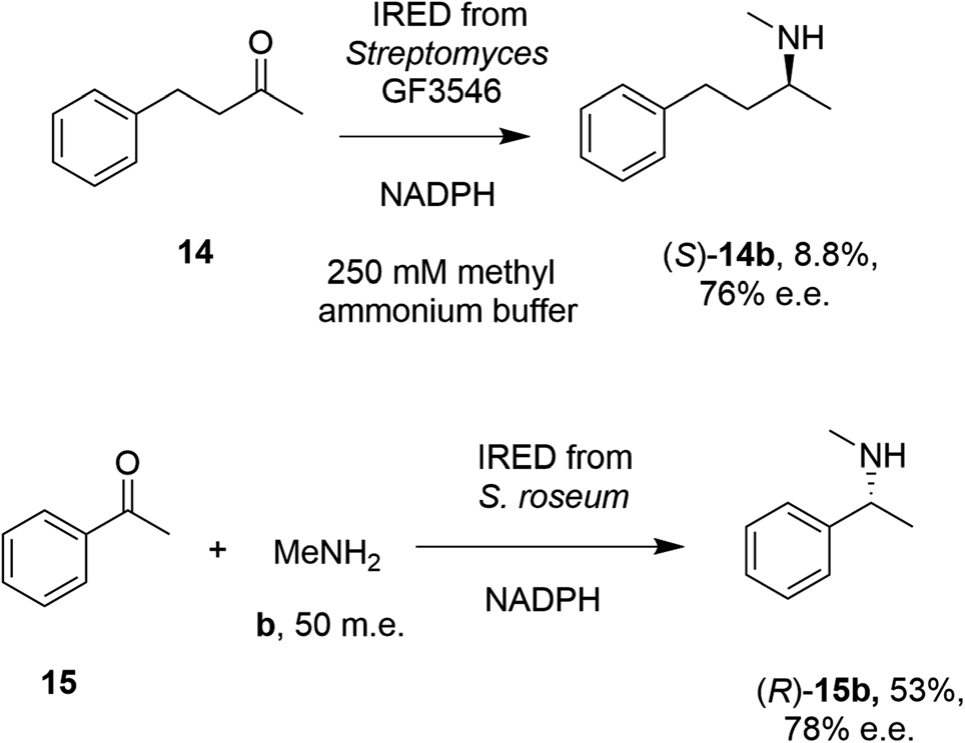
Early examples of intramolecular asymmetric reductive amination reactions enabled by IREDs at high amine: ketone ratios.^49,55^ (m.e. = molar equivalents). ‘NADPH’ in this and other schemes signifies the requirement of this reduced cofactor for the reaction, and is usually generated with the assistance of a cofactor recycling system comprising *e.g.* glucose dehydrogenase (GDH) and glucose, omitted from this and other schemes.

In 2014, Müller and co-workers first established that the IRED from *Streptomyces* GF3546, if challenged with 20 mM 4-phenyl-2-butanone 14 in a reaction medium containing 250 mM methylammonium buffer, representing a 12.5 molar equivalents (m.e.) of amine, gave a low conversion (8.8%) to the secondary amine product (*S*)-2-(methylamino)-4-phenylbutane 14b (Scheme 4).^49^ However, a good enantiomeric excess of 72% demonstrated the principle that a prochiral imine, recruited from solution, could be bound and reduced asymmetrically by an IRED. Further work by Nestl^55^ consolidated this discovery by showing that further IREDs, such as the (*R*)-IRED-*Sr* from *Streptosporangium roseum*, would catalyze the reductive amination of ketones including acetophenone 15, with small amine partners such as methylamine b, when provided at 50 m.e., to give the chiral secondary (*R*)-amine product 15b with 53% conversion and 78% e.e. (Scheme 4). Interestingly, NMR studies were unable to detect imine formation in solution for this reaction, suggestive of the ability of the IRED to rapidly draw the imine from solution for reduction.

**Scheme 5:**
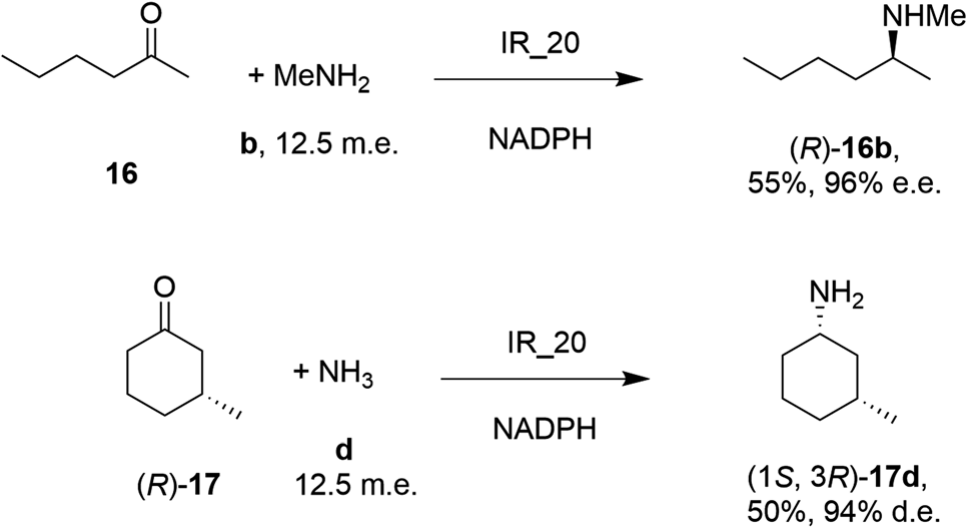
Screening of an IRED library by Roche identified IR_20 as a candidate enzyme for enabling reductive aminations of 16 and (*R*)-17 to form 16b and (1*S*,3*R*)-17d respectively.^56^

Researchers at Roche published the first survey of an industrial library of IREDs applied to reductive amination reactions (Scheme 5).

**Scheme 6:**
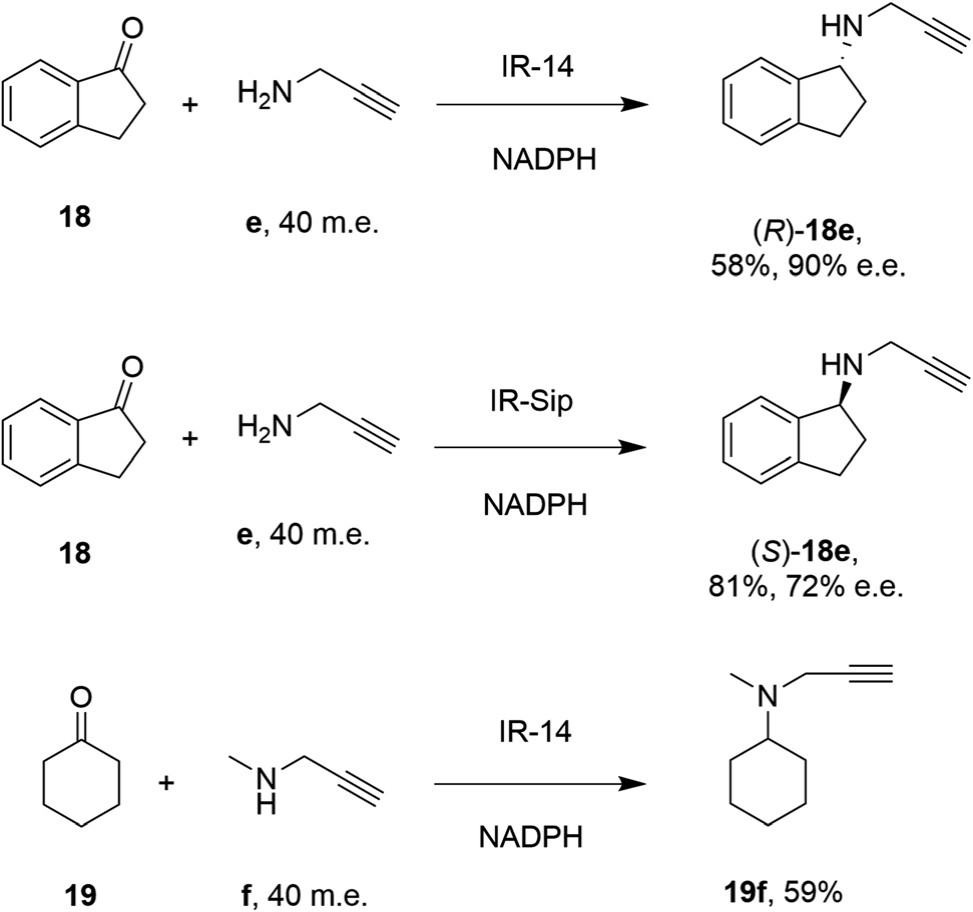
IREDs from the Roche library catalyzed the synthesis of both enantiomers of rasagiline 18e and tertiary amine 19f.^57^

28 enzymes were screened for their ability to enable the amination of model ketones including acetophenone, cyclohexanone and 2-hexanone with ammonia, methylamine and butylamine.^56^ Amines were supplied at 12.5 m.e. over the ketone substrates. Reductive aminations with methylamine were found to be the most effective, but in this case acetophenone was shown to be a poor substrate, with no enzymes yielding conversion >10%. However, aliphatic ketones (2-hexanone 16 and cyclohexanones) could be coupled successfully with methylamine b. The IR_20-catalyzed reductive amination of 2-hexanone 16 with methylamine b was scaled up to 400 mg, offering (*R*)-amine product 16b in 55% isolated yield and 96% e.e. Similarly, the reductive amination of (*R*)-3-methylcyclohexanone 17 with ammonia d using IR_20 gave a 50% yield of (1*S*,3*R*)-amine product 17d with 94% d.e. Matzel and co-workers, working with the Roche collection of IREDs, then demonstrated the synthesis of the anti-Parkinson's agent (*R*)-rasagiline 18e directly from indanone 18 (Scheme 6) and propargylamine e, if the latter were supplied in large excess.^57^

**Scheme 7:**
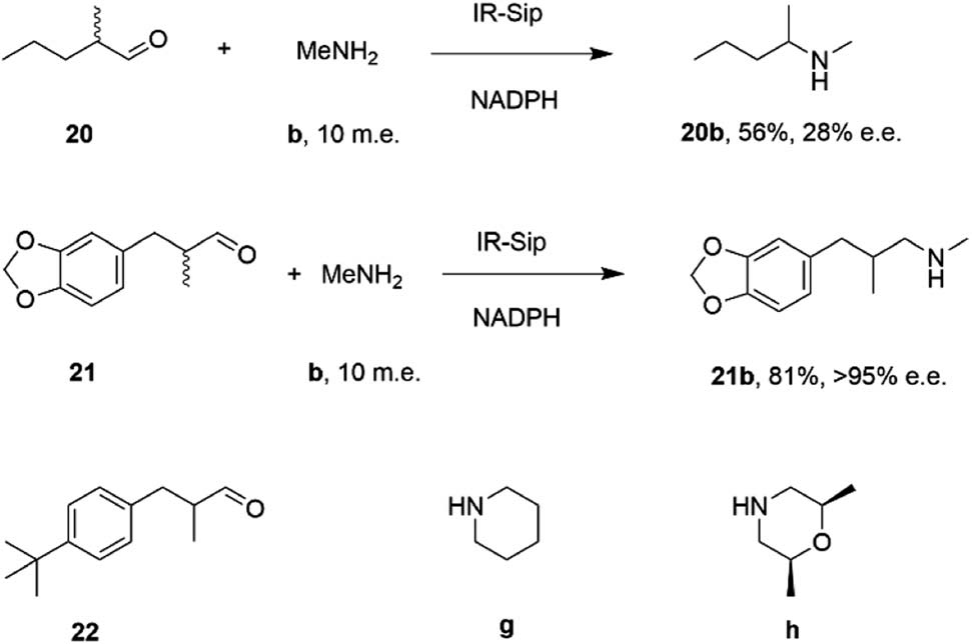
IR-Sip catalyzed amination of α-racemic aldehydes.^58^

IR-14 was also applied to the 100 mg scale amination of 10 mM 18, with 40 m.e. of amine e, to give the product (*R*)-18e in 58% isolated yield and with 90% e.e. The enantiocomplementary enzyme IR-Sip gave (*S*)-18e in 81% yield and 72% e.e. under similar conditions. IR-14 also enabled the reductive amination of cyclohexanone 19 with 40 m.e. of *N*-methyl propargylamine f, the first secondary amine accepted in IRED-reductive amination to give product 19f, in 59% yield (Scheme 6). The same group also showed that IREDs could mediate-dynamic kinetic resolution reactions during the reductive amination of racemic α-branched aldehydes, which are liable to racemise under aqueous reaction conditions, leading to the synthesis of enantioenriched β-chiral amines.^58^ Hence when IR-Sip was presented with 20 mM *rac*-20 or 21 and 10 m.e. of methylamine, 20b or 21b were prepared in 56% yield and 28% e.e. or 81% yield and >95% e.e. respectively (Scheme 7). The reductive amination of aldehyde 22 with amines g and h in pursuit of the (*S*)-configured fungicides febropidine and fenpropimorph proved more challenging, giving only 9% and 6% conversion respectively. This low turnover was attributed to steric constraints within the active sites of the selected IREDs.

These encouraging observations prompted investigation into the role of IREDs in the catalysis of the reductive amination reaction. It was clear that reduction must be achieved in the enzyme once the prochiral imine was formed, but there was little evidence about the role of the enzyme in imine formation as conversions and reaction rates at low amine:ketone ratio were reported to be low.

## Reductive aminases (RedAms)

**Scheme 8:**
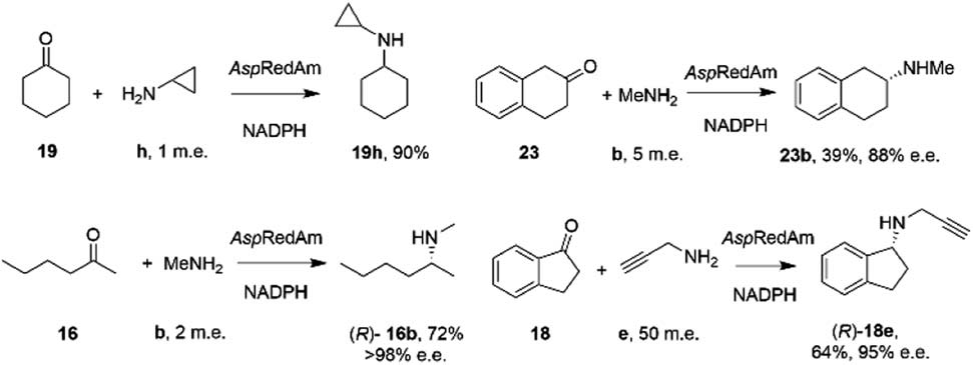
Reductive amination reactions catalyzed by the reductive aminase from *Aspergillus oryzae* (*Asp*RedAm).^38^

By analogy with abiotic asymmetric reductive amination, IRED-catalyzed reductive amination would be more synthetically attractive if amine m.e.s could be reduced, ideally to stoichiometric equivalency with the carbonyl substrate. In 2017, Aleku and co-workers reported the discovery of an (*R*)-selective IRED from the filamentous fungus *Aspergillus oryzae* (*Asp*RedAm), which displayed the ability to catalyze the reductive amination of a broad selection of ketones with amines including cyclohexanone 19 methylamine b, propargylamine e and cyclopropylamine i (Scheme 8).^38^

In contrast to previous studies, *Asp*RedAm-catalyzed reductive amination could be performed when the enzyme was provided with cyclic or C6 aliphatic ketones and only one m.e. of a small hydrophobic amine partner, giving near quantitative conversion to the corresponding secondary amines.^38^ However, less activated ketones, such as indanone 18, required higher m.e. of amine for high conversion. This study raised for the first time the possibility that the enzyme was, in some cases, catalyzing not only the reduction of an imine recruited from solution, but also the formation of the imine from ketone and amine. This subclass of IREDs were termed ‘Reductive Aminases’ (RedAms). Notably, *Asp*RedAm catalyzed the reductive amination of hexanal with allylamine at pH 7.0 and 9.0 at similar rates, which was in contrast with other IREDs, for which a 20-fold increase in activity was observed under basic conditions, in which imine concentration in solution is more pronounced. This observation further suggested that *Asp*RedAm was able to catalyze imine formation as part of the reductive amination process. Kinetic measurements established that the enzyme employed a ter–bi mechanism, analogous to that employed by *N*-methyl l-amino acid dehydrogenase,^59^ in which cofactor, then ketone, then amine, are bound sequentially, followed by release of the secondary amine product and finally the oxidized cofactor. To illustrate the preparative potential of the reactions, *Asp*RedAm was applied to the 100 mg scale amination of 50 mM 19 with methylamine b using 2 m.e. to give the hydrochloride salt in 75% isolated yield.

## Structure and mechanism

**Fig. 3:**
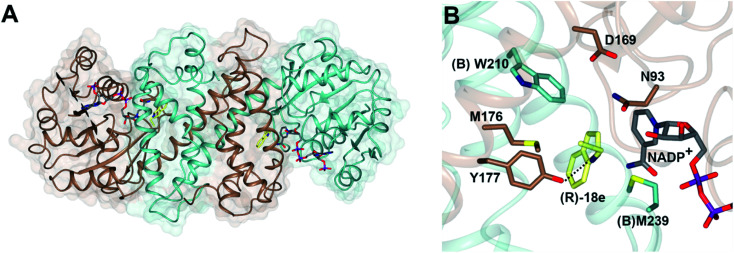
(A) Structure of dimer of *Asp*RedAm (5 G6S) with monomers (A) and (B) in brown and blue respectively; (B) active site of *Asp*RedAm with NADP^+^ and (*R*)-rasagiline (*R*)-18e.^38^ Residues are from monomer (A) unless marked as (B). The interaction between the nitrogen atom of 18e and the side chain of Y177 is indicated by a black dashed line.

The structure of *Asp*RedAm (Fig. 3A) was determined in the presence of both the cofactor and (*R*)-rasagiline 18e and again revealed the accommodation of the ligand within the closed hydrophobic pocket of the active site, with the electrophilic carbon in close proximity to the C-4 atom of NADP^+^ (Fig. 3B).^38^ The enzyme featured a pendant aspartate D169 in the active site, which, when mutated to alanine or asparagine, resulted in mutants with a 200-fold reduction in reductive amination activity. In addition, the amine group of the ligand made close contact with the side chain of tyrosine Y177, possibly indicating a role for this residue in either substrate anchoring or proton donation to the nascent amine. Mutation of Y177 also resulted in a 32-fold decrease in catalytic efficiency (*k*_cat_/*K*_m_).

The structure of *Asp*RedAm also permitted the first rational mutations to be conducted in pursuit of variants with altered activity. Mutation of (B)W210, at the back of the active site as pictured in Fig. 3B, to alanine, gave (B)W210A, which displayed an inverted stereopreference for the amination of 2-tetralone 23 with propargyl amine e to form product (S)-23e with 80% e.e., compared to 95% (*R*)- for the wild-type enzyme.

The discovery of *Asp*RedAm prompted the study of further homologs of the enzyme in a bid to unearth complementary or improved activity, and also further models for structural and mechanistic studies.^60^ A homolog from *Aspergillus terreus* (*At*RedAm) proved to be reliable with respect to crystallization, and a number of complex structures was achieved.

**Fig. 4:**
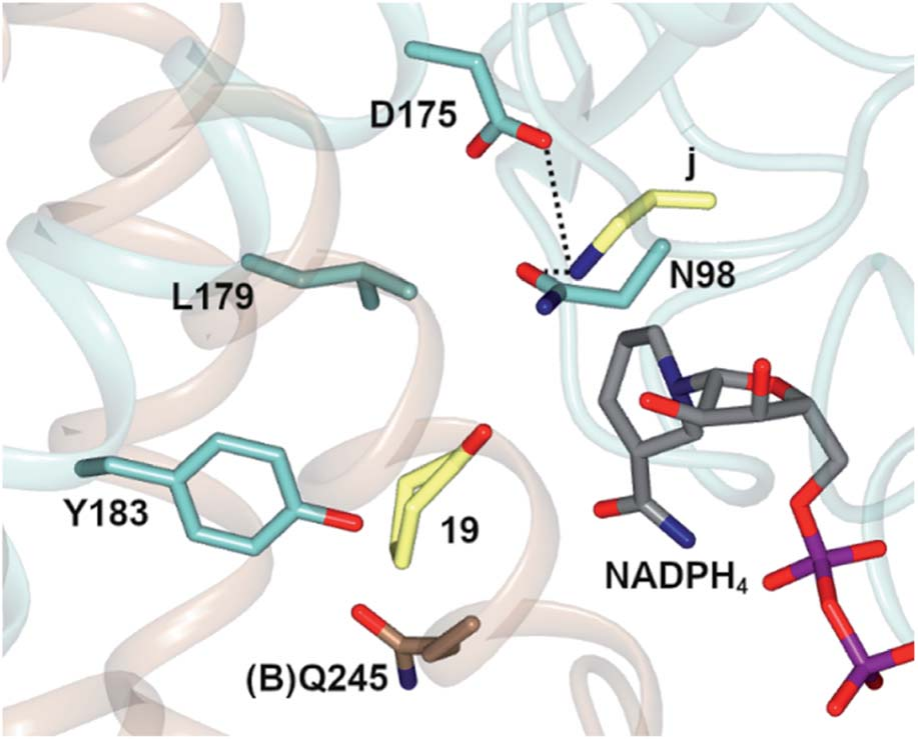
Active site of *At*RedAm in complex with redox-inactive cofactor NADPH_4_, cyclohexanone 19 and allylamine j.^60^ Residues are from monomer (A) unless marked as (B). Interactions between j and the side chains of D175 and N98 are shown as black dashed lines.

In one, the enzyme was crystallized in the presence of cyclohexanone 19, allylamine j and the redox-inactive cofactor NADPH_4_, the use of which appeared to arrest the reductive amination process in the enzyme (Fig. 4).

The nitrogen atom of allylamine was shown to interact with the side chains of both D175 and also N98, and the cyclohexanone carbonyl to be in the proximity of the phenolic hydroxyl of Y183. Mutation of D175 to alanine resulted in a mutant D175A, for which activity could not be measured. Mutation of Y183 to phenylalanine resulted in a mutant Y183F for which the *K*_m_ for 19 was increased 6-fold.

**Scheme 9:**
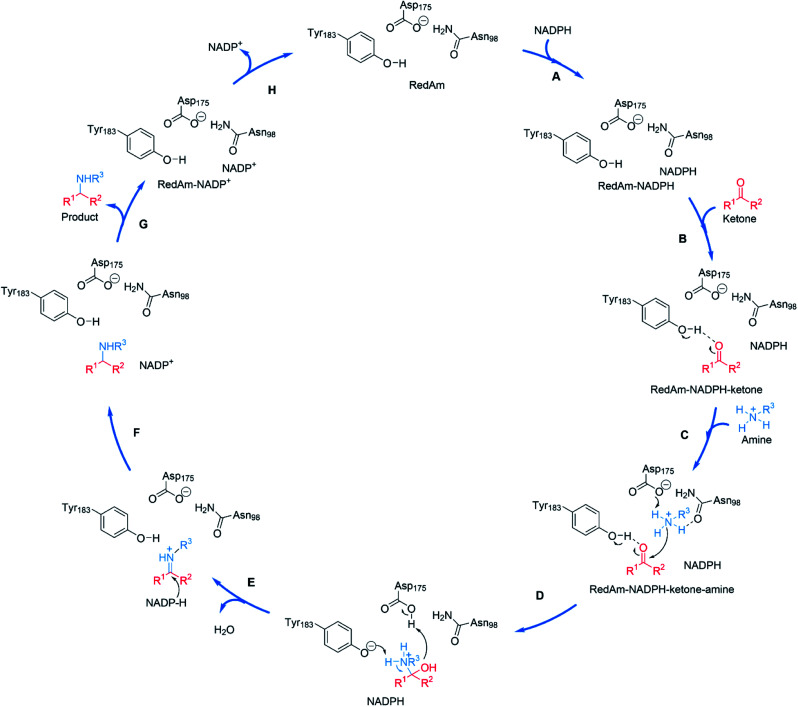
Proposed mechanism for a fungal RedAm-catalyzed reductive amination using residue numbering from *At*RedAm.^60^ (A) The active site of *At*RedAm binds NADPH, (B) cyclohexanone, and (C) the amine sequentially, to form a quaternary complex; (D) the amine is activated by D175 for attack at the electrophilic carbon of the ketone to give a hemiaminal intermediate; (E) the hemiaminal intermediate loses water to give the iminium intermediate; (F) this is reduced by NADPH to give the amine product and NADP^+^; (G) the amine product and (H) NADP^+^ are sequentially expelled from the active site to regenerate the enzyme for another catalytic cycle.

The structural and mutational data permitted a mechanism for the fungal RedAm-catalyzed amination of ketones with small hydrophobic amines to be postulated (Scheme 9).^54^ Following binding of the reduced cofactor, the ketone was bound within the active site of the RedAm, with the carbonyl group secured by the hydroxyl of a tyrosine residue Y183. Following this, the small amine bound and was activated for attack at the carbonyl by D175, in combination with N98. Both Y183 and D175 residues are conserved in fungal RedAms that were the subject of this study, but it is clear that many IREDs with reductive aminase activity possess different residues at these key positions, notably Y in the case of D175, and W in the case of Y183, although no mutation or complex structural data has yet been presented to clarify the roles of those residues in relevant enzymes. Indeed the variety of amino acids within IREDs that appear to catalyze reductive amination reactions suggests that a number of variations on this model will exist.

## Applications

The discovery of the IRED-catalyzed reductive amination instigated further applications of the reaction and also studies into enzyme homologs that may catalyze reactions with complementary substrate range and selectivity.

**Scheme 10:**
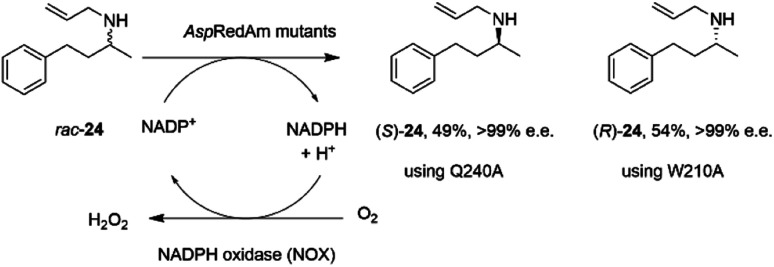
Resolution of racemic amine 24 by *Asp*RedAm.^55^

It was first demonstrated that, in addition to the quantitative formation of chiral secondary amines from ketones, fungal RedAms, such as *Asp*RedAm, would also catalyze the *resolution* of chiral secondary amines if supplied with the oxidized cofactor NADP^+^, at the elevated pH of 10.5.^61^ Hence the Q240A variant of *Asp*RedAm catalyzed the resolution of racemic *N*-allyl-4-phenylbutan-2-amine 24 to give 49% conversion to amine (*S*)-24 with >99% e.e., using NADPH oxidase (NOX) to recycle the cofactor (Scheme 10).

In addition, the W210A mutant of *Asp*RedAm, which often displayed complementary enantioselectivity, catalyzed the resolution of the same compound to give the (*R*)-24 product, again with 54% conversion and >99% e.e.

**Scheme 11:**
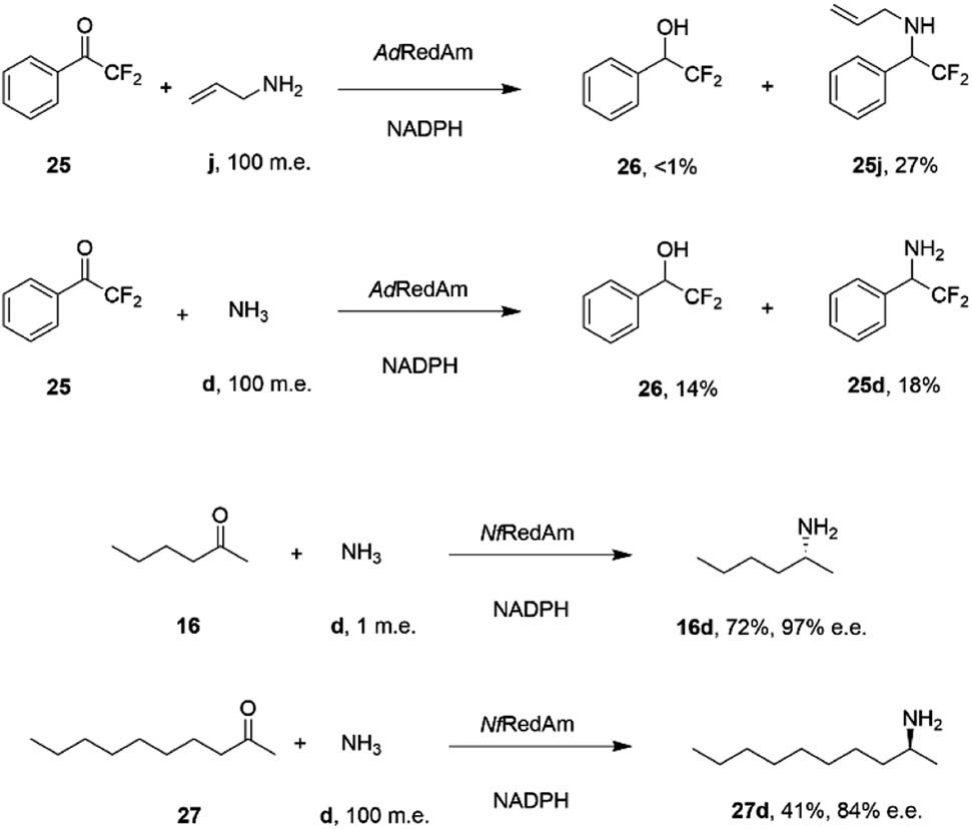
Reductive aminations catalyzed by RedAms from *Neosartorya* spp.^63,64^

The catalytic promiscuity of the fungal RedAms extended to the amination of fluoroketones. Catalysis by IREDs is, for the most part, distinguished by their inability to reduce ketones to alcohols, except in the case of highly activated substrates such as 1,1,1-trifluoroacetophenone.^62^ González-Martínez and co-workers studied this phenomenon with respect to a number of fungal RedAms and mono, bi- and trifluorinated ketone substrates with small amines.^63^ It was determined that, while the expected amines were produced, the proportion of alcohol product increased with the number of fluorine substituents, but this was also dependent upon the nature of the amine partner. Hence α,α-difluoroacetophenone 25 was transformed by *Ad*RedAm to a mixture of alcohol 26 and amine 25j when allylamine j was the donor, but to a much higher proportion of alcohol 26 and amine 25d with ammonia (Scheme 11).

This result was attributed to the competition for binding of the ketone and relevant imine intermediate in each case. The superior ability of some fungal RedAms to enable reductive aminations using ammonia was further investigated by Mangas-Sanchez and co-workers.^64^ When challenged with 10 mM cyclohexanone and only one m.e. of ammonia, RedAms from the fungi *Neosartorya fumigatus* (*Nf*RedAm) and *Neosartorya fischeri* (*Nfis*RedAm) gave 90% and 81% conversion respectively. Interestingly, while *Nf*RedAm catalyzed the amination of 2-hexanone 24 to give (*R*)-2-aminohexane 24d with 72% conversion and 97% e.e. (Scheme 11), a switch in stereoselectivity was observed for 2-decanone 27, with (*S*)-27d being obtained with 41% conversion and 84% e.e. with the addition of 100 m.e. of NH_4_Cl. The *Neosartorya* RedAms were also observed to be more thermotolerant than other fungal RedAms, with only a small reduction in activity following incubation at 50 °C for 60 min. *Nf*RedAm was applied to the amination of 2-hexanone 16 at a substrate concentration of 80 mM (8 g L^−1^), to give a space-time-yield (STY) of 0.34 g L h^−1^ of 16d, but this performance was greatly improved by immobilization of the enzyme on an EziG resin, which resulted in continuous production of (*R*)-2-aminohexane 16d with an STY of 8.1 g L h^−1^.

Reductive amination activity was also described for IREDs from outside the realm of fungi. Researchers at Pfizer published a study of 45 IRED enzymes from bacteria, in which their capacity for the reductive amination of model ketones with amine partners was evaluated.^65^ In addition to IREDs displaying activity with known substrates, the study was successful in identifying enzymes with some affinity for bulkier ketone and amine partners. Hence IR91 catalyzed the amination of 2-tetralone 23 with methylamine b to give (*S*)-23b in 74% conversion and >98% e.e., whereas IR46 gave the (*R*)-enantiomer. IR44 catalyzed the amination of cyclohexanone with benzylamine, aniline and pyrrolidine with 79%, 11% and 99% conversion respectively at 10 mM ketone and 2.5 m.e. of amine. The amination with pyrrolidine was performed on a 625 mg scale at a substrate concentration of 125 mM, giving a 71% isolated yield of *N*-cyclohexylpyrrolidine. Interestingly, little conversion was observed with ammonia as the amine partner.

**Scheme 12:**
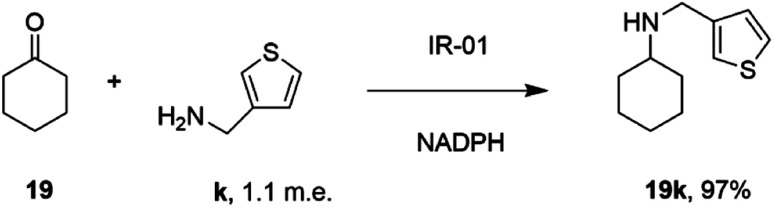
The formation of melanin-concentrating hormone receptor agonist 19k using GSK IR-01.^66^

In a similar fashion, researchers at GSK assembled a library of 85 IREDs from diverse sources, and again tested their abilities to catalyze reductive aminations of cyclohexanone 19, 4-fluorophenylacetone, and aromatic aldehydes, with bulkier amines including aniline and benzylamine, but also amino thiophenes and aminopyridines, each provided at only 1.1 m.e.^66^ As part of this screen, IR-01 was reported to catalyze the amination of cyclohexanone 19 with 3-thienylmethylamine k to form *N*-(thiophen-3-ylmethyl)cyclohexanamine 19k, a precursor of a melanin-concentrating hormone receptor agonist, with 97% conversion (Scheme 12).

The performance of IR-01 was further evaluated for the amination of cyclohexanone with substituted anilines, showing broad selectivity with *meta*- and *para*-substituted rings, especially those with electron donating groups. Interestingly, IR01 also exhibited selectivity in the reductive amination of cyclohexanone using benzene-1,4-diamine, giving a 99 : 1 ratio of monoaminated to diaminated products.

Montgomery and co-workers at Johnson-Matthey formulated a library of 95 IREDs that included targets from both fungal and plant genomes.^67^ As in the Pfizer and GSK studies above,^65,66^ enzymes were identified that were competent for the reductive amination of cyclohexanone 19 with bulkier amines, such as benzylamine, phenethylamine and aniline, provided at only 2 m.e. Three enzymes, p-IR06, p-IR-17 and p-IR-79 gave >60% conversion for the amination with benzylamine. The conversion of 50 mM cyclohexanone 19 with 100 mM aniline by p-IR89 was improved through the incorporation of co-solvents, notably cyclohexane, when added as a 200 μL layer to a 700 μL reaction, giving a conversion of 81%.

**Scheme 13:**
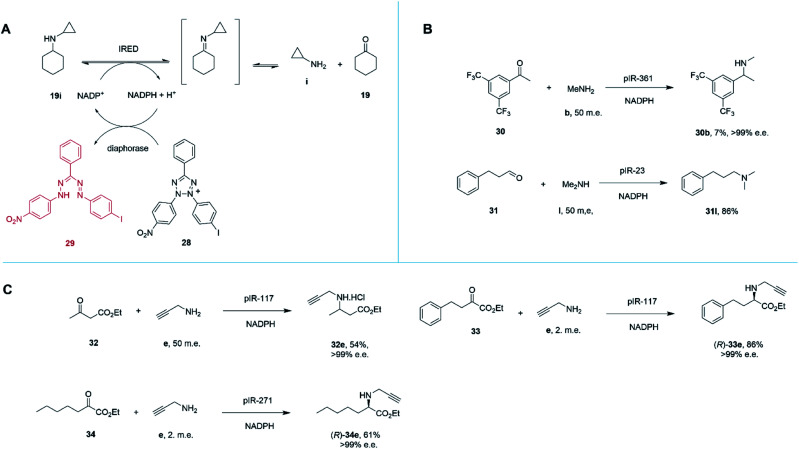
(A) Chemical basis of IRED-y-to-go screen.^68^ The oxidative activity of an IRED, acting on a secondary amine substrate, generates NADPH, which is used by diaphorase to reduce tetrazolium salt 28 to red dye 29. (B) The IRED-y-to-go screen was used to identify IREDs that enabled aminations of acetophenones 30 and the production of tertiary amines.^68^ (C) The IRED-y-to-go screen enabled also identified enzymes for the amination of esters.^68,69^

Marshall and co-workers recently presented an extensive survey of 384 IRED sequences which, unlike the industrial libraries detailed above, were largely identified from metagenomic samples.^68^ This study was also successful in addressing the high-throughput evaluation of IRED reductive aminase activity, which is complicated by the range of possible ketone and amine partners. This was accomplished through the development of a colorimetric screen (IREDy-to-go) that could be applied and read in multi-well plates, in which the NADPH generated from the oxidative activity of IREDs in the presence of amine substrates, is used by a diaphorase to reduce tetrazolium salt 28, leading to the formation of a red formazan dye compound 29 (Scheme 13A). The expressed genes were evaluated for their catalysis of a number of reductive amination reactions, revealing a diversity of activity with respect to imine reduction *versus* reductive amination, activity and stereoselectivity. In addition to known transformations, the survey was especially revealing in identifying IREDs with superior activity for ‘difficult’ substrates such as acetophenone, than had been observed previously.

For example, pIR-361 catalyzed the amination of 3,5-ditriflouromethylacetophenone 30 with methylamine b to give amine 30b, a precursor of the NK1 receptor antagonist orvepitant, with >99% e.e., albeit with 7% conversion, (Scheme 13B). pIR-23 catalyzed the amination of hydrocinnamaldehyde 31 with dimethylamine l to give the tertiary amine product 31l with 86% conversion (Scheme 13B). Selected IREDs were also identified that catalyzed the amination of β-keto esters with small amines to give *N*-substituted β-amino esters. In this mode, eight IREDs were identified and pIR-117 was applied to the synthesis of 32e from ethylacetoacetate 32 and propargylamine e (Scheme 13C), with an isolated yield of 54% and >99% e.e.^68^

This library of metagenomic IREDs also furnished catalysts for the reductive amination of α-keto esters to form *N*-substituted α-amino esters.^69^ Screening against the model substrate 25 mM ethyl-2-oxo-4-phenylbutyrate 33 with 2 m.e. of propargylamine e, 78 and 20 enzymes from the library were found to be (*R*)- and (*S*)-selective respectively. pIR-117 catalyzed the synthesis of (*R*)-33e with 86% conversion and >99% e.e., but when the substrate had a methyl substituent, the (*S*)-product with 97% e.e. was obtained (Scheme 13C). Preparative scale reactions were performed for selected enzymes and substrates, with pIR-271 used to accomplish the reductive amination of 34 to give product (*R*)-34e with 61% isolated yield and >99% e.e.

**Scheme 14:**
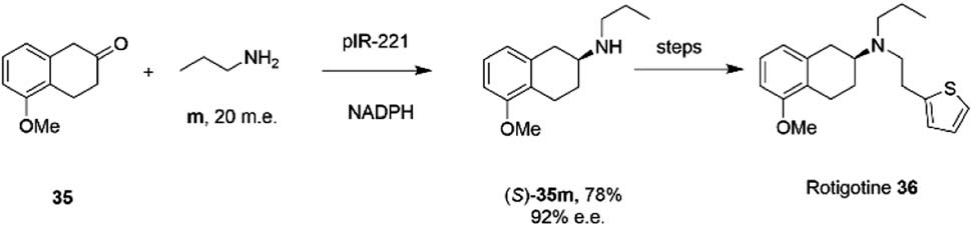
Synthesis of anti-Parkinson's agent (*S*)-rotigotine 36.^70^

The library of metagenomic IREDs was further evaluated for the reductive amination of bicyclic 2-ketotetralin and 2-ketochroman derivatives by Citoler and co-workers.^70^ Screening of the panel with 5 mM ketone, and either 100 or 200 m.e. of amine, identified enzymes pIR-88 and pIR-221 that successfully aminated 5-methoxy-2-tetralone 35 with *n*-propylamine m to give the secondary amine product 35m with conversions of 87% and 92% and enantiocomplementary (*R*)- and (*S*)- products with >99% and 92% e.e. respectively. pIR-221 was applied to the 1 mmol scale synthesis of 35m at 20 mM concentration, giving a 78% isolated yield of (*S*)-35m with 92% e.e., which could then be further elaborated by *N*-alkylation and demethylation over two steps to give the anti-Parkinson's agent (*S*)-rotigotine 36 (Scheme 14).

**Scheme 15:**
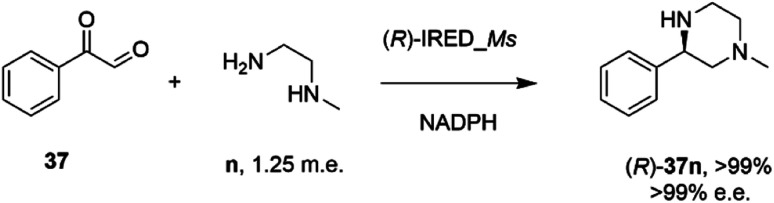
Intramolecular reductive amination for the preparation of piperazines.^71^

In an elegant extension to the reductive amination reaction, Nestl and co-workers showed that IREDs could be applied to the synthesis of piperazines from diamine and dicarbonyl substrates through a double reductive amination.^71^ The ‘*R*-IRED_*Ms*’ enzyme from *Myxococcus stipitatus* catalyzed the formation of (*R*)-1-methyl-3-phenyl piperazine 37n with >99% conversion and >99% e.e. when the enzyme was incubated with 5 mM and 1.25 m.e. of the diamine n and dicarbonyl substrate 37 respectively (Scheme 15).

Incubation of 5,6-dimethyl-2,3-dihydropyrazine with the enzyme gave no products, suggesting that the reactive pathway was likely to proceed *via* a condensation–reduction–condensation–reduction, rather than by two condensations followed by two reductions, one of which would constitute an intramolecular reductive amination. The substrate scope was expanded to 2,3-diamines and 1,4-dicarbonyl substrates with a variety of alkyl substituents tolerated. The production of (*R*)-1-methyl-3-phenyl piperazine 37n was scaled up to 50 mM substrate concentration, giving 8.1 g L^−1^ of the product in 87% isolated yield.

**Scheme 16:**
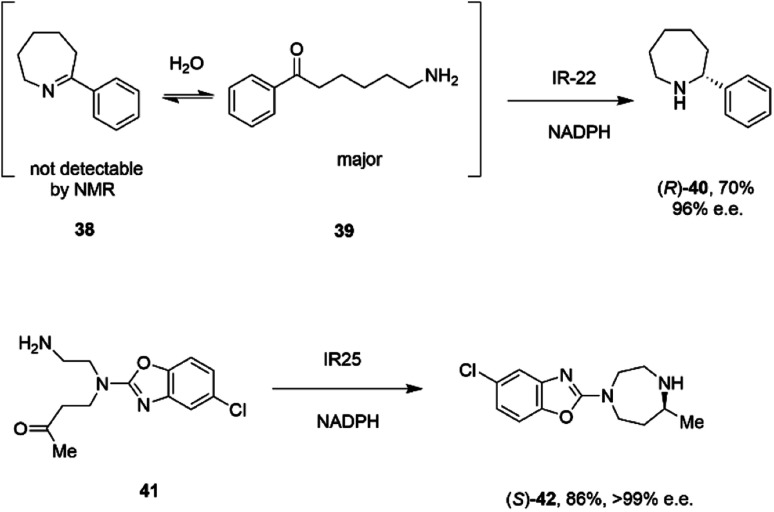
Intramolecular reductive aminations for the formation of azepanes.^72,73^

A study of the synthesis of 2-substituted azepanes by IREDs revealed a further possible intramolecular reductive amination reaction catalyzed by these enzymes.^72^ While a straightforward asymmetric reduction of seven-membered ring imine 38 might be envisaged, this species is not detectable in solution by NMR, suggesting that equilibrium favours the ring-opened amino ketone 39 (Scheme 16).

Hence, the IRED IR-22 was used to catalyze the intramolecular reductive amination of the compound to (*R*)-2-phenylazepane 40 with 70% conversion and 90% e.e. Additional IREDs were employed in the reductive amination of related substrates, with *Cf*IRED from *Cystobacter ferrugenius*, giving the (*S*)-series of products and the metagenomic IRED mIRED10 giving the (*R*)-enantiomers. This phenomenon of intramolecular reductive aminations was further investigated by Zhu and co-workers, who screened a library of 48 IREDs for the enantioselective formation of 1,4-diazepanes,^73^ through cyclisation of substrates such as 4-((2-aminoethyl)(5-chlorobenzo[*d*]oxazol-2-yl)amino)butan-2-one 41 (Scheme 16), a precursor to the insomnia treatment Suvorexant. Activity for the formation of both enantiomers of the product was detected in multiple ‘IRED-fold’ IREDs, but the best enzyme, then selected for improvement by structure-guided mutagenesis, was actually a pteridine reductase ‘IR1’ from *Leishmania major*, an enzyme involved in folate metabolism, which synthesized the sought after (*R*)-enantiomer of the product in 99% e.e. The activity extended to more standard IREDs, such as IR25 from *Micromonospora echinaurantiaca*, which catalyzed formation of the complementary (*S*)-product. IR25 was applied to the 100 mL scale transformation of 100 mM 41 to give the product (*S*)-42 in 86% yield and with >99% e.e.

## Applications in cascades

The ready accessibility of IREDs, coupled with progress in the production and application of other enzymes, meant that the enzyme-catalyzed reductive amination reactions were ideally suited to cascade reactions for the elaboration of simple and cheap precursors, notably hydrocarbons and alcohols, to secondary amines. The feasibility of applying IREDs in multi-enzyme cascades had been established in a carboxylic acid reductase (CAR)-ω-TA-IRED cascade for the conversion of carboxylic acids to cyclic amines.^74^ The reductive amination capability of IREDs could also be applied to the synthesis of secondary amines if ketone substrates could be generated *in situ*.

In a first application, it was realized that NADPH-dependent RedAms might be combined with NADP^+^-dependent alcohol dehydrogenases (ADHs) to convert racemic alcohols to chiral amines in one pot with closed loop cofactor recycling – a hydrogen-borrowing cascade.^75^ In this mode ADH-150 was combined with *Asp*RedAm in the one-pot oxidation-amination of 5 mM cyclohexanol to *N*-propargyl-cyclohexylamine with 91% conversion using 1 mM NADP^+^ and 50 m.e. of propargylamine. A combination of a further ADH, the W110A mutant ADH from *Thermoanaerobacter ethanolicus* (*Te*SADH) and *Asp*RedAm, was successful in converting racemic 1-(4-fluorophenyl)-2-propanol to the (*R*)-amine product with 41% conversion and 95% e.e. ADH-150 and *Asp*RedAm were finally combined in a 100 mg scale conversion of cyclohexanol to *N*-allylcyclohexylamine in 61% yield.

**Scheme 17:**
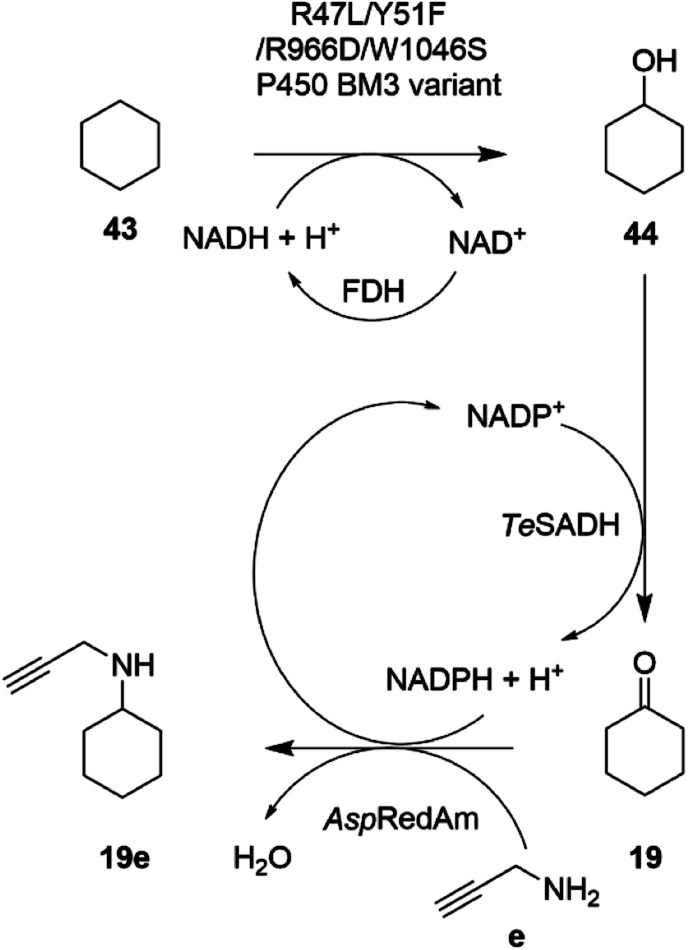
Enzyme cascade for the amination of cyclohexane.^76^ FDH = formate dehydrogenase.

In an extension to this study, Tavanti and co-workers showed that cyclohexane 43 could be successively hydroxylated, to cyclohexanol 44, oxidized to cyclohexanone 19 and then aminated using a cascade of the R47L/Y51F/R966D/W1046S mutant of cytochrome P450BM3, the W110A mutant ADH from *Thermoanaerobacter ethanolicus* (*Te*SADH) and *Asp*RedAm.^76^ The choice of enzymes permitted the closed-loop recycling of reducing equivalents from NADPH for the RedAm as *Te*SADH used NADP^+^ as cofactor (Scheme 17).

A mixture of enzyme lysates transformed cyclohexane, provided neat to form a two-phase system, to 4.5 mM *N*-methylcyclohexylamine, but conversions were negatively affected by inhibition of the P450 by the amine donor, and also the competition of endogenous *E. coli* enzymes for reducing equivalents. Hence, a two-step, one pot approach was adopted, in which hydroxylation was completed prior to the addition of ADH, RedAm and amine, resulting in 19.6 mM of *N*-propargylcyclohexylamine 19e. A 20 mL scale two-step reaction was performed that yielded 50 mg of the product.

**Scheme 18:**
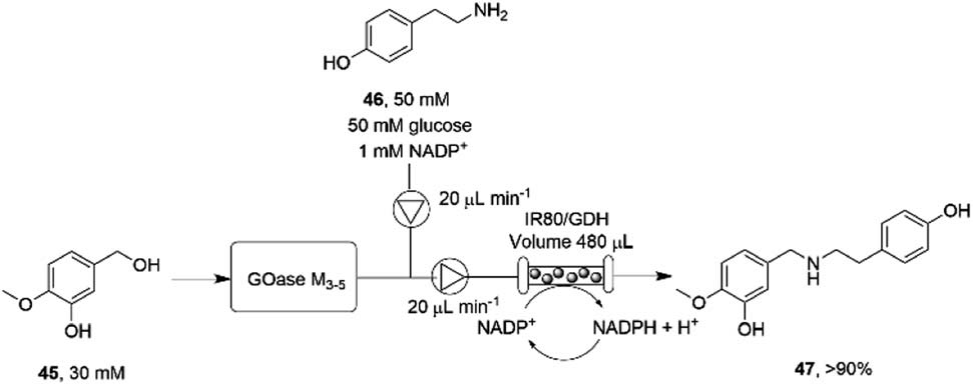
Flow system for the oxidation-reductive amination cascade towards the synthesis of 4-*O*-methyl norbelladine 47.^77^

The necessity for separation of biocatalytic steps in the last example is illustrative of the lack of compatibility between some systems that militates against the construction of one-pot cascades. Mattey and co-workers have investigated continuous flow systems in an effort to separate otherwise incompatible systems, and applied these to reductive amination cascade reactions.^77^ A multipoint injection reactor (MPIR) was employed in which an immobilised choline oxidase mutant first generated aromatic aldehydes from alcohol substrates. The product was then fed into a reactor containing the immobilised RedAm from *Ajellomyces dermatitidis* (AdRedAm) and a glucose dehydrogenase for NADPH recycling. This gave a flow system that produced an STY of 2.1 g L^−1^ h^−1^ for 4 h. In a further elaboration, the amine donor for the RedAm reaction could be generated by a ω-TA, with both immobilized ω-TA and RedAm kept in separate packed bed reactors to stop cross reactivity with amines. In this mode, aldehydes such as butyraldehyde and benzaldehyde could be generated using the ω-TA and then fed to the RedAm reactor with cyclohexanone, giving STYs of the secondary amine products of up to 2.53 g L^−1^ h^−^1. The flow cascade was applied to the synthesis of the pharmaceutical precursor 4-*O*-methyl norbelladine 47 (Scheme 18) from isovanillyl alcohol 45, which was first oxidized to isovanillin using an immobilised galactose oxidase variant (GOase M_3–5_) in the first reactor. The intermediate aldehyde was then fed to the second reactor containing the RedAm IR80, along with amine donor tyramine 46, to synthesize the product 47 with an STY of 2.26 g L^−1^ h^−1^ over a 4 h period.

**Scheme 19:**
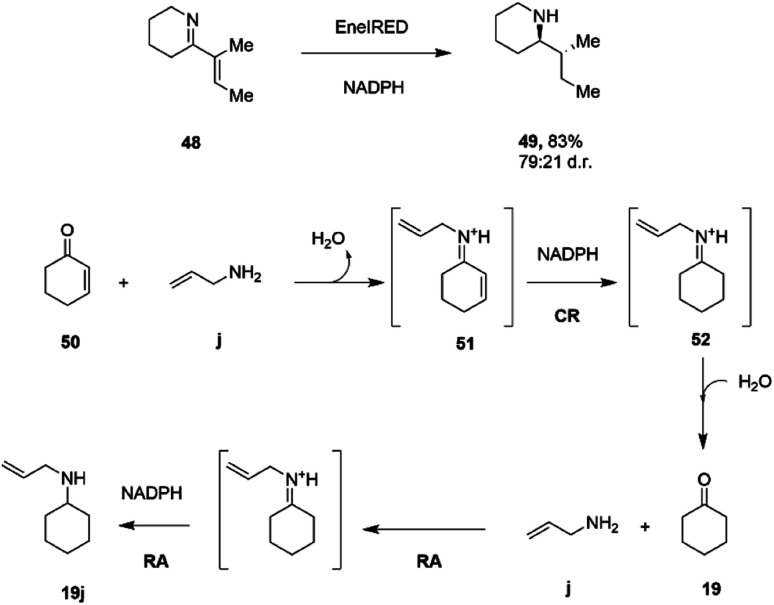
Conjugate Reduction (CR) – Reductive Amination (RA) catalyzed by EneIRED.^79^

A further cascade process combined the successive C=C bond reduction catalyzed by ene-reductases (EREDs) with the IRED mediated reduction of C=N bonds, to convert cyclic ene–imines to amines with two chiral centres.^78^ However, it did not prove possible to perform both reactions in one pot, as the IRED reaction was much faster than that of the ERED, and the allylic amines formed were not substrates for the latter enzyme. However, a subsequent screen of the ene–imine substrates against IREDs alone unearthed a surprising promiscuous activity of some enzymes for the reduction of both C=C and C=N bonds.^79^ A screen of 389 IREDs revealed that 44 of the enzymes were competent for the four-electron reduction of the model ene–imine compound 48 (Scheme 19) to the saturated amine 49 with a particular enzyme, termed EneIRED, catalyzing the reaction with 83% conversion and a diastereomeric ratio of 79 : 21. An extension to this observation was that EneIRED also catalyzed the C=C bond reduction in cyclohex-2-enone 50 followed by reductive amination using allylamine j, giving *N*-allylcyclohexylamine 19j with 69% isolated yield, constituting a conjugate reduction-reductive amination reaction (CR-RA).^79^ No CR products were formed in the absence of the amine substrate, strongly indicating a mechanism whereby the substrate for CR is the condensation product 51 of unsaturated ketone and amine (Scheme 19). Following reduction of 51 using NADPH, release of the intermediate 52 into solution would result in hydrolysis to the saturated ketone, which would then be bound by the IRED, along with the amine, in the active site with NADPH to achieve a reductive amination to give secondary amine product 19j. The CR-RA catalysis by EneIRED extended to a range of acyclic and cyclic enones and also enals, in which double bonds were reduced and intermediates reductively aminated with small amines to give products in up to 77% isolated yield. The dual activity of IREDs in this mode suggests that further complex multi-electron reductions catalyzed by these enzymes might be achieved in the future.

## Scalable reactions and industrial examples

A Design of Experiments (DoE) approach was taken to the optimization of a model IRED catalyzed reductive amination reaction at scale.^80^ Dominguez and co-workers employed a customized factorial design, followed by principal component analysis, to the study of the reductive amination of cyclohexanone 19 with cyclopropylamine i. Initial measurements indicated that the concentration of ketone and amine, and the stability of the IRED enzymes used for the study, were the most significant parameters in reaction optimization. Initial reactions using 200 mM 19 and 2 m.e. of the amine in batch reactions gave 87% conversion to the product using IRED-69, although attempts to increase substrate concentration were not successful with this system. Conversions and yields at higher substrate concentration could be achieved using a fed-batch system in which concentrations of up to 750 mM ketone, supplied at 100 mM h^−1^, were converted quantitatively to the amine product 19i using IRED-33, with a turnover number of 48 279 and an STY of 12.9 g L^−1^ h^−1^.

**Scheme 20:**
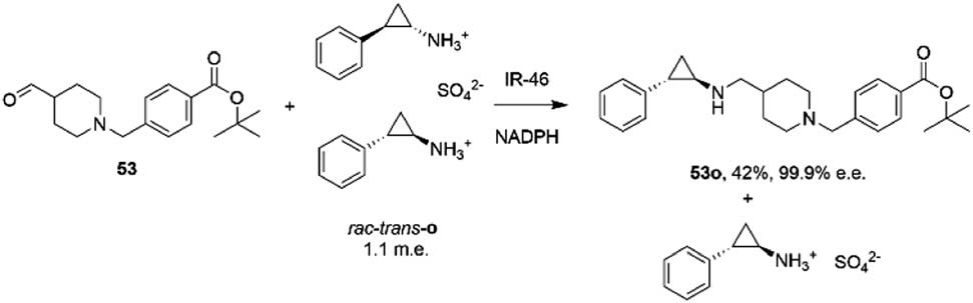
Synthesis of lysine-specific demethylase-1 (LSD) inhibitor GSK2879552 intermediate 53o by reductive amination with IR46.^39^

The first published use of extensive protein engineering for the optimisation of an IRED-catalyzed reductive amination was published by researchers at GSK.^39^ In this study, the target was the coupling of the aldehyde 53 with bulky amine tranylcypromine sulfate o, with the desired product being the (1*R*,2*S*)- product 53o (Scheme 20), an intermediate for the synthesis of the lysine-specific demethylase-1 (LSD) inhibitor GSK2879552.

**Fig. 5:**
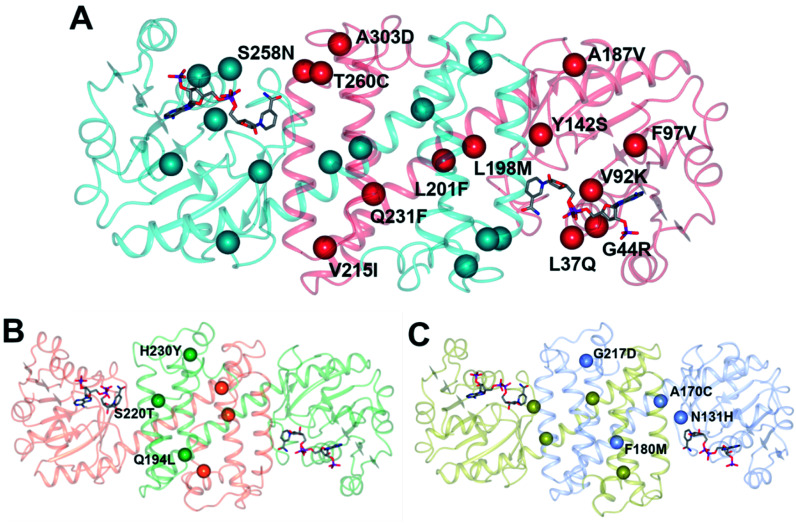
(A) Model of structure of ‘IR-46’ derived using *Ao*IRED (5FWN).^39^ The model shows the location of amino acid residue sites that were mutated in variant ‘M3,’ applied in the kg-scale synthesis of 53n in Scheme 20; (B) structure of IRED88 (ref. 81) (PDB 7OG3) showing locations of residues mutated in variant Q194L/S220T/H230Y, used in the gram-scale synthesis of 54b in Scheme 21; (C) model of structure of *Sp*RedAm^40^ showing locations of residues mutated in variant ‘R3-V6’ used in the ton-scale synthesis of 58b (Scheme 22).

An initial screen of GSK IREDs for the reaction using 2.2 m.e. of the racemic amine identified ‘IR-46’ from *Saccharothrix espanaensis* as the superior enzyme, which gave the product with 42% isolated yield and 99.9% e.e. in a 5 g scale reaction. The enzyme was targeted for evolution for process suitability, especially for lower pH, to assist reactant and product solubility, but also for improvements in substrate and biocatalyst loading. 256 out of 296 possible residues were subjected to site saturation mutagenesis (SSM), and mutants screened at a substrate concentration of 12 g L^−1^. Mutant Y142S (‘M1’) was identified as the superior mutant under these conditions, with conversion improved 40-fold compared to the wild-type enzyme. A model of IR-46 suggested that the amino acid side-chain at position 142 makes an H-bond with the pendant aspartate D194 in the active site. Combined with information on other sites at which beneficial mutations were identified, a combinatorial library of 4000 variants based on the first round was further assessed, and yielded the multiple-site mutant ‘M2’ (Y142S, L37Q, A187V, L201F, V215I, Q231F, S258N) with mutation sites at different locations within the IRED (Fig. 5A). Modelling again suggested that, although some mutations were within or near the active site, and may have roles in substrate positioning (L198M, L201F) or cofactor interactions (G44R, V92K), some were more likely to have roles in improving hydrophobic interactions that stabilised the active IRED dimer.

A further round of mutation yielded mutant ‘M3’ (Y142S; L37Q, A187V, L201F, V215I, Q231F, S258N; G44R, V92K, F97V, L198M, T260C, A303D), which displayed a 38 000-fold improvement in turnover number compared to the wild-type (wt), and was applied in process conditions to the 20 L scale manufacture of the amine product to give 1.4 kg of 53o from three runs with 84.4% yield and 99.7% e.e. Interestingly, a study of both melting temperature and half-life of the enzyme showed the improved process performance of mutant ‘M3’ was largely due to increases in these parameters, which were improved by 30 °C and 500-fold respectively over the wild-type, while specific activity was increased only 13-fold.

**Scheme 21:**
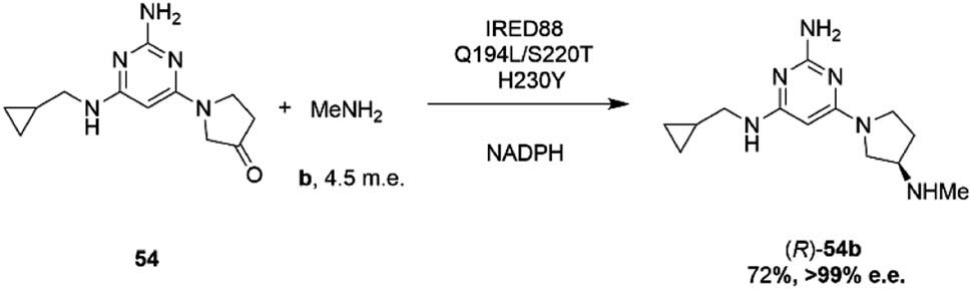
Synthesis of ZPL389 54b using enzyme IRED88 Q194L/S220T/H230Y evolved by Novartis.^81^

Researchers at Novartis have combined genome mining with recent advances in machine learning for the evolution of an IRED for the stereoselective reductive amination of ketone 54 with methylamine b to molecule ZPL389 54b (Scheme 21), an H4 receptor antagonist used in the treatment of atopic dermatitis.^81^ Library screening plus genome mining was used to identify the enzyme ‘IRED88’ as being able to catalyze the amination of 54 to the product 54b with 70% conversion, and to the required (*R*)-enantiomer, but with only 30% e.e. A deep mutation scanning (DMS) approach was taken first, in which a library of mutants composed of all amino acid positions mutated to the 19 alternative residues was screened for activity by mass spectrometry. This revealed a mutant S220T, at the mouth of the active site cleft, which now gave 96% e.e., an improvement that was attributed to restricted access to the active site for the pro-*S* conformer of the substrate. Three rounds of error prone PCR (epPCR) using S220T as the platform gave mutant Q194L/S220T/H230Y with 85% conversion and 99% e.e. The locations of these mutations are shown in Fig. 5B. Applying these data to machine learning algorithms suggested mutants which, when expressed, gave lower e.e.s. However, when machine learning was coupled with structure-guided evolution, superior mutants were identified, including M129L/A156S/Y177W, which gave 59% conversion and 93% e.e. The epPCR generated variant Q194L/S220T/H230Y was selected for a 1 g scale reaction in which a 72% yield of the product was obtained at a substrate concentration of 100 mM, and with >99% e.e. Further analysis of this variant determined that it had a markedly increased melting temperature of 57 °C *versus* 42 °C for the wild-type, and two-fold the specific activity. This study led to several interesting conclusions about IRED engineering: machine learning was useful as a ‘sample efficient’ strategy to suggest variants with improved properties, which can each serve as a basis for more focused mutation strategies. In any case, it was important that hits were validated using larger scale reactions. The study also showed that a combination of improvements in both specific activity and stability were desirable, and also that linear additivity was observed for mutant loci outside the active sites, but that this phenomenon was more difficult to predict for active site mutations, where cooperativity between amino acids involved in the mechanism of stereoselectivity was likely.

**Scheme 22:**
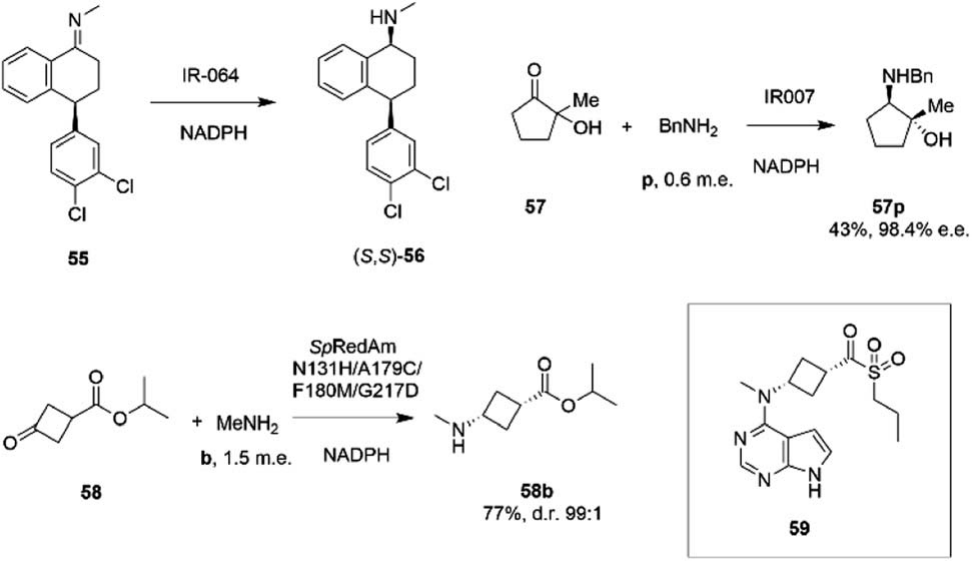
Reductive amination reactions investigated by Pfizer.^40,82,83^

Researchers at Pfizer also instigated a programme of work directed at the application of IREDs to industrial reductive amination reactions. Following their description of a family of enzymes with a range of activity,^65^ they then reported the application of enzyme IR_064 from *Myxococcus fulvus* to the production of sertraline using an imine reduction.^82^ In this work, the enzyme catalyzed the enzymatic reduction of the precursor ((*E*)-4-(3,4-dichlorophenyl)-*N*-methyl-3,4-dihydronaphthalen-1(2*H*)-imine) 55 to make (*S*,*S*)-sertraline 56 (Scheme 22).

This was followed up by a study of a reductive amination reactions focusing on the synthesis of a key intermediate for an inhibitor of the oncology target CDK/2/4/6.^83^ 88 IRED enzymes were screened for the ability to catalyze the kinetic resolution and reductive amination of 200 mM racemic hydroxyketone 57 with 120 mM benzylamine hydrochloride p to give secondary amine product 57p (Scheme 22). The enzyme IR007, identified as the best candidate, was subjected to directed evolution, to give a final variant IR007-143 of superior activity, giving 43% conversion from a substrate concentration of 50 g L^−1^. A 35% isolated yield from the racemate with 98.4% e.e. was achieved. A cofactor recycling system using glucose dehydrogenase was applied, with sodium hydroxide to maintain the pH of 7.0, as the system results in the formation of gluconic acid. Developments in RedAm engineering were consolidated at Pfizer with the recent development of a RedAm-catalyzed process for the production of a precursor of the Janus Kinase 1 (JAK1) inhibitor abrocitinib 59 (Scheme 22).^40^ 80 IREDs were initially screened for their ability to catalyze the amination of isopropyl (1*S*,3*S*)-3-(methylamino)cyclobutane-1-carboxylate 58 with methylamine b to form the *cis*-isomer of the desired product 58b (Scheme 22). An IRED from *Streptomyces purpureus* (*Sp*RedAm) was identified as the best candidate and applied to the 7.5 g scale reactions at a substrate loading of 20 g L^−1^, giving the product with 27% isolated yield and a d.r. of >99 : 1. *Sp*IRED was therefore selected for protein engineering, starting with site saturation mutagenesis at 93 sites of 296 possible residues, based on homology models and bioinformatics analysis. The most improved variants, for which mutational sites were at or near the active site, gave >75% conversion at 50 g L^−1^ substrate. A second round of evolution combined some of the beneficial mutants to give double and triple variants, of which N131H/A170C and A170C/F180M were selected for further rounds, with position 170 further from the active site. A multi-site random recombination approach was applied to the best mutants to give variants with 4 to 6 mutations of improved activity at substrate loadings of 75 g L^−1^. Further recombination in a 3rd round gave variant ‘R3-V6’ (N131H, A170C, F180M, G217D) (Fig. 5C) of which only position 180 was within the active site, with the others located in the 2nd shell. The final variant, which exhibited an improvement in activity of 206-fold was applied to the synthesis of the product on an increasingly large scale, from batch (1–10 kg) to pilot (50–100 kg) to manufacturing (>200 kg), with 77% conversion reported, against 0.75% for the wt under process conditions. Using this procedure 3.5 MT of the product was synthesized.

## Conclusions

The development of IRED-catalyzed asymmetric reductive aminations from laboratory discovery to industrial scale application illustrates the rapid progress that can now be made with biocatalytic reactions once useful transformations have been identified. In this particular case, the process has been accelerated by the availability of a wide range of contemporary techniques in various disciplines that are now available as established tools for enzyme improvement. Hence, following enzyme discovery and initial biochemical and structural characterisation, informed choices of homologs as starting points for enzyme programs can be made either through screening database homolog sequences or metagenomic resources, using rapid cloning technology and the design of appropriate HT screens. Following this, medium or high-throughput mutation of targets for process improvement can advance initial hits quickly, and data that result from these experiments can now be fed into machine-learning algorithms for the identification of the most useful improvements, and in some cases, in the prediction of further beneficial mutations. These studies have already illustrated that, in the case of IREDs and RedAms, considerations of the process suitability of biocatalysts, including thermostability, tolerance of co-solvents and half-life, can be as important as mutations that directly affect substrate recognition or turnover. In the case of reductive aminations, although many advances have been made, many challenges remain. The basis for improvements in activity has been difficult to characterize, as structural data on candidate enzymes in complex with their target molecules is difficult to acquire. This difficulty is compounded by a lack of understanding of the nature of the enzymatic process that is being catalyzed. A continuum appears to exist between true reductive aminations, catalyzed by RedAms, and typified by the reaction of small activated ketones such as cyclohexanone and small hydrophobic amines, and imine reductions, where pre-formed imines are rapidly recruited from solution for asymmetric reduction. More specific information will be required on each target reductive amination therefore, in order for improvements in ketone, amine, and imine recognition to be better targeted, providing more robust platforms for enzyme redesign.

## Author contributions

All authors contributed to the writing of the manuscript.

## Conflicts of interest

There are no conflicts to declare.

## Supplementary Material
